# A cross‐sectional study on the association between residential noise exposure, cognitive functioning, and structural markers of brain damage ‐ The Maastricht Study

**DOI:** 10.1002/alz70860_104278

**Published:** 2025-12-23

**Authors:** Jens Soeterboek, Kay Deckers, Walter H. Backes, Jacobus F.A. Jansen, Miranda T. Schram, Hans Bosma, Jeroen Lakerveld, Manu Sastry, Sebastian Köhler

**Affiliations:** ^1^ Mental Health and Neuroscience Research Institute (MHeNs), Maastricht University, Maastricht, Limburg, Netherlands; ^2^ Alzheimer Center Limburg, Maastricht University, Maastricht, Netherlands, Maastricht, Limburg, Netherlands; ^3^ School for Mental Health and Neuroscience, Maastricht University, Maastricht, Netherlands; ^4^ Alzheimer Center Limburg, Mental Health and Neuroscience Research Institute, Maastricht University, Maastricht, Netherlands; ^5^ Department of Radiology, Maastricht University Medical Center+, Maastricht, The Netherlands, Maastricht, Limburg, Netherlands; ^6^ Mental Health and Neuroscience Research Institute, Maastricht University, Maastricht, Netherlands; ^7^ School for Cardiovascular Diseases, Faculty of Health, Medicine and Life Sciences, Maastricht University, Maastricht, Netherlands; ^8^ Department of Radiology, Maastricht University Medical Center+, Maastricht, The Netherlands, Maastricht, Netherlands; ^9^ CARIM School for Cardiovascular Diseases, Maastricht University, Maastricht, Netherlands; ^10^ Department of Internal Medicine, Cardiovascular Research Insitute CARIM, Maastricht University, Maastricht, Netherlands; ^11^ Heart and Vascular Centre, Maastricht University Medical Centre, Maastricht, Netherlands; ^12^ Department of social Medicine, Maastricht University, Maastricht, Netherlands; ^13^ Care and Public Health Research Institute, Maastricht University, Maastricht, Netherlands; ^14^ Institute for Risk Assessment Sciences, Utrecht University, Utrecht, The Netherlands, Utrecht, Utrecht, Netherlands; ^15^ Department of Epidemiology and Data Science, Amsterdam Public Health Research Institute, Amsterdam UMC, VU University Amsterdam, Amsterdam, Netherlands; ^16^ Academic sleep centre Ciro, Horn, Netherlands; ^17^ Mental Health and Neuroscience Research Institute (MHeNs) and European Graduate School of Neuroscience (EURON), Faculty of Health, Medicine and Life Sciences (FHML), Maastricht University, Maastricht, Limburg, Netherlands; ^18^ Alzheimer Center Limburg, Maastricht University, Maastricht, Limburg, Netherlands

## Abstract

**Background:**

Environmental noise exposure is known to negatively affect both physical and mental health through various pathways. Given the heterogeneous findings in previous research, further investigation into the impact of environmental noise on brain health and cognitive function is crucial. This study explores the relationship between both objective and subjective residential noise exposure and cognitive functioning, as well as markers of brain damage, in a large adult population. The analysis also considers dementia risk factors and demographic variables for a comprehensive understanding.

**Method:**

Cross‐sectional data on cognitive functioning and brain volumes (MRI markers) from 4,018 Dutch participants aged 40‐75 from The Maastricht Study were matched with address‐level noise exposure data (noise: from roads, railways, aviation, industry, and wind turbines) from the Geoscience and Health Cohort Consortium. Subjective noise exposure (traffic, work, and neighbor noise) was assessed via self‐report questionnaires. Linear and non‐linear relationships were explored using multiple regression analyses and restricted cubic splines, adjusted for air pollution, demographic and dementia risk factors. Pathways via hearing loss, daytime sleepiness, and depression were also assessed through interaction analyses.

**Result:**

Higher residential noise exposure was linked to lower white matter (WM) volume and higher cerebrospinal fluid (CSF) volume. An U‐shaped relationship was observed between residential noise and grey matter (GM) volume. Objective noise exposure was not associated with cognitive function. Subjective noise exposure at work was associated with worse overall cognition, memory, and executive functioning and attention. Interaction analyses for hearing loss, daytime sleepiness, and depression did not alter the results. Sensitivity analyses revealed a significant positive interaction between objective noise and age for CSF volumes.

**Conclusion:**

Our findings indicate that higher residential noise exposure is associated with brain atrophy (lower WM and higher CSF volumes), while the relationship with GM was unexpected. Additionally, subjective noise exposure at work was linked to worse cognitive performance, suggesting a discrepancy between objective and subjective noise effects. Lastly, older individuals might be more vulnerable to the effects of noise exposure. Future research should explore factors such as housing quality, noise mitigation, and the potential threshold effects of noise exposure on brain health.

